# Experimental Validation of Falling Liquid Film Models: Velocity Assumption and Velocity Field Comparison

**DOI:** 10.3390/polym13081205

**Published:** 2021-04-08

**Authors:** Ruiqi Wang, Riqiang Duan, Haijun Jia

**Affiliations:** Institute of Nuclear and New Energy Technology, Tsinghua University, Beijing 100085, China; qiqiD22@163.com (R.W.); Jiaha@tsinghua.edu.cn (H.J.)

**Keywords:** falling liquid films, PIV/PLIF, SPLIF, PTV, WRM

## Abstract

This publication focuses on the experimental validation of film models by comparing constructed and experimental velocity fields based on model and elementary experimental data. The film experiment covers Kapitza numbers Ka = 278.8 and Ka = 4538.6, a Reynolds number range of 1.6–52, and disturbance frequencies of 0, 2, 5, and 7 Hz. Compared to previous publications, the applied methodology has boundary identification procedures that are more refined and provide additional adaptive particle image velocimetry (PIV) method access to synthetic particle images. The experimental method was validated with a comparison with experimental particle image velocimetry and planar laser induced fluorescence (PIV/PLIF) results, Nusselt’s theoretical prediction, and experimental particle tracking velocimetry (PTV) results of flat steady cases, and a good continuity equation reproduction of transient cases proves the method’s fidelity. The velocity fields are reconstructed based on different film flow model velocity profile assumptions such as experimental film thickness, flow rates, and their derivatives, providing a validation method of film model by comparison between reconstructed velocity experimental data and experimental velocity data. The comparison results show that the first-order weighted residual model (WRM) and regularized model (RM) are very similar, although they may fail to predict the velocity field in rapidly changing zones such as the front of the main hump and the first capillary wave troughs.

## 1. Introduction

Falling liquid films are examples of open-flow systems that bring a laminar steady state to a disordered state in both time and space as the film develops [1,2]. Film flows have a long wave nature, the typical length of which is much larger than the thickness of the film. These features enable modelling of the flow with reduced equations from the Navier–Stokes equations; however, the models need solid experimental data to be validated and verified [1,3,4].

To validate these different models, knowledge of the velocity field with the correlating film topology under various surface waves would be of great value [5]. High-quality velocity field data with correlating film interfaces also have the following benefits: (i) velocity field data make it possible to calculate the transfer processes in flowing films [6]; (ii) they provide essential testing of the accuracy of models in the nonlinear regime by evaluating the accuracy of their velocity field predictions under waves [1]; and (iii) can directly validate basic assumptions applied in different models.

Experimentally, the hydrodynamic characteristics of falling liquid films have been investigated vigorously in the past, especially film thickness, which indicates the interface wave status of film flows. Various experiments have attempted to measure velocities in falling liquid films since the 1950s, and the applied techniques include hot wire anemometry [7], stereoscopic photography [8], laser Doppler anemometry [9,10,11], high-speed video photography [12,13], particle image velocimetry (PIV) [14,15,16,17,18], and particle tracking velocimetry (PTV) [15]. However, most of the previous investigations of the velocity field in wavy film flows, either time-averaged or instantaneous data, were compared to the parabolic profile, which is a theoretical model given by Nusselt for steady and flat film flows. Even for the experiments stated in the context above, only some experimentally measured spatial-temporal velocity field data within transient wavy falling films are available. The low spatial or temporal resolution also limits the further application of experimental velocity fields.

This paper focuses on the high spatial resolution and temporal resolution of experimental velocity field measurements in transient film flows, which will be beneficial for further improving the velocity field measurement capacity in a falling film on an inclined plate, allowing for quantitative velocity field research in films with submillimeter thickness, mostly corresponding to large Kapitza number liquids such as water.

The remainder of this article is structured as follows: in Section 2, the experimental facility, including the film test section, flow loop, PIV/PLIF system and film experiment cases, is briefly described. In Section 3, the experimental methodology is illustrated, and additional methodological validations are given, including Nusselt’s validation with PIV/PTV and an adaptive PIV method validation. In Section 4, data analysis is performed, mainly focusing on a transient velocity field comparison. In Section 5, conclusions are drawn.

## 2. Experimental Facility

### 2.1. Experimental Film Test Section and Flow Loop

**Figure 1:**
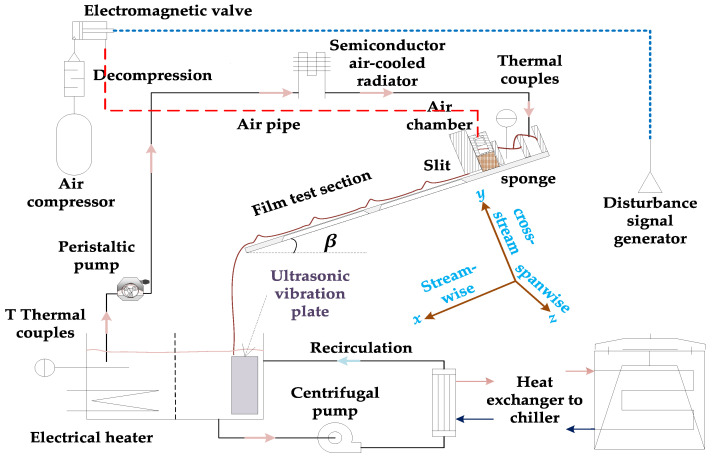
Schematic diagram of the experimental flow loop and film test section. The italic letters *x*, *y*, and *z* denote the streamwise, cross-stream, and spanwise directions of the film description coordinate system, respectively.

**Figure 2:**
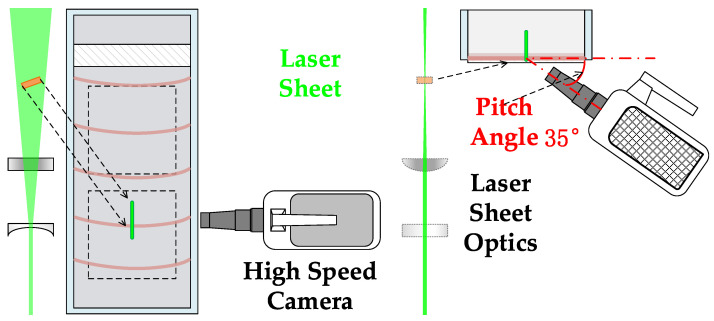
For the film test section, high-speed camera and laser sheet, the high-speed camera is set up beside the test section, as illustrated in the figure. The left part and right part is the same test section of a high speed camera and laser sheet, just being given in different views, in which the left is from the film cross-stream direction, and the right is from the streamwise direction. The high speed camera’s pitch angle against the film spanwise direction is set to 35 degrees.

A short description of the film test section and flow loop is provided in Figure 1 and Figure 2. More details can be found in the work of Wang [19]. The principal component of the film test section is a 670 × 220 × 0.7 mm^3^ soda–lime glass plate that serves as the film substrate and a polymethyl methacrylate (PMMA) box. The measurements in this article were taken 350 mm downstream of the slit and center in the spanwise direction.

Fluid in the flow loop is pumped with a peristaltic pump, which could provide an accurate but pulsed small flow rate. There is a flow distribution part in the film test section buffering and dispensing the fluid into a pulsation-free flow. The flow loop has an additional recirculating loop, electrical heater, and ultrasonic plate, which could maintain constant fluid temperature and particle concentrations. Additionally, disturbances are provided with compressed air regulated by an electromagnetic valve, and the disturbance frequency is controlled by a digital square wave.

### 2.2. PIV/PLIF System

To acquire a 2D planar *x*–*y* cross section of the liquid film flow on an inclined plate, which has quasi-2D characteristics, with macroscale spanwise dimensions (0.2 m) and a microscale cross-section (approximately 0.5 mm), high optical magnification is required to properly resolve the streamwise velocity profile on the millimeter scale of the film cross section. Moreover, the image acquisition accessible method would be limited below the glass plate. The camera and lens are set in an off-axis setup, and the imaged plane should be determined by the thin laser sheet to image the velocity field perpendicular to the glass plate. A high recording rate is required to properly resolve the film hydrodynamics due to its transient nature.

To tune the normal 2D PIV system applicable for the stated velocity field and topology measurement, the adopted experimental measurement technique is PIV/PLIF, which combines PIV with PLIF by using a single camera to simultaneously acquire the velocity and topology.

An adequate number of particles is the primary concern for the fidelity of velocity measurements, because the measurement technique determines the velocity of tracer particles instead of the velocity of the fluid [20]. The particles used are 2 μm titanium dioxide particles. The working fluids in the experiment are also doped with florescent dye (rhodamine 6G). The additional fluorophore could radiate florescence photons of longer wavelengths after excitation. For PIV/PLIF image recording, no filter is added in front of the lens, and both the particle-scattered light and florescence are recorded. The latter could provide the film topology to distinguish the liquid film from the air or the glass plate.

To satisfy the requirements of high optical magnification, a large depth of field and an adequate working distance, the chosen lens was an ultra-macro lens (Anhui Changgeng Optics Technology, LAOWA ULTRA MACRO lens, Hefei, Anhui, China). This lens can provide 2.5–5-fold optical magnification with a working distance of approximately 40–45 mm. Unlike usual microscopy, an ultra-macro lens can provide an independent small aperture, and the lens work has a large depth of field, which ensures that the whole film region can be imaged even if the focused plane does not coincide with the desired film cross-section. A detailed image of the acquisition setup can be found in Figure 2. The lens and camera were mounted on two-axis goniometers that were mounted on three-dimensional positioning stages.

**Figure 3:**
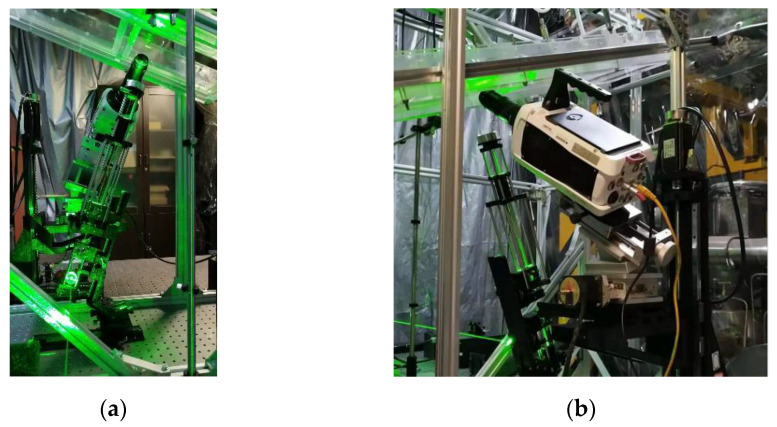
Photographs of PIV/PLIF measurement experiments. (**a**,**b**) were photos corresponding to schematic diagrams in Figure 2.

The image plane position ambiguity was caused by the large depth of field and the off-axis setup of the lens, which was compensated for by customized thin laser sheet illumination. The laser source was a continuous wave (CW) Nd:YAG 532 nm laser from Beijing Ranbond Technology Co., Ltd., Beijing, China. It had a maximum power of 6 W. The beam laser was shaped via a plano-concave cylindrical lens (f = −25.4 mm) diverging the laser into a planar sheet, and a plano-convex cylindrical lens (f = 100 mm) reducing the beam waist diameter. As shown in Figure 3, the laser optics were accommodated with an optical cage system using four rigid steel rods to ensure a common optical axis and better mobility. In the experiments, the cage system was mounted on a three-dimensional manual translation stage for precise positioning and focusing.

Finally, the illuminated film cross section (*x*–*y* plane in Figure 1) was recorded with a high-speed camera (Vision Research, model Phantom V711, Wayne, NJ, USA).

When used in structured planar laser induced fluorescence (SPLIF) mode, a long-pass filter was mounted in front of the lens, and a structured glass plate was added to shadow the light sheet into parallel structured illumination. The long-pass filter (LOPF-25C-561 from OptoSigma, Tokyo, Japan) provides a high transmittance range wavelength of 571.1–900 nm with over 93% transmittance. The structured plate was a customized target (glass substrate with vacuum-sputtered chrome, laser carved 50 mm × 50 mm and 5 lines per millimeter in a Ronchi rulings pattern) to form structured illumination.

The developed single-camera PIV/PLIF implementation, the first of its kind to the best of the authors’ knowledge, avoids the alignment and registration issues associated with dual-camera PIV/PLIF combinations [21]. In addition, the CW laser is continuous and stable, while the dual-pulsed laser has an alignment issue: dual-pulse PIV lasers do not perfectly overlap [22], and one frame might not be in the same focus as the other frame, causing a relatively low depth of field compared to a normal lens. Moreover, with the ultra-macro lens option, there would not be enough room below the film glass plate for two high-speed cameras together with the lens properly positioned. Ultimately, the high frame rate, which could be used to resolve the transient wavy film flow field, motivated our development of a single-camera PIV/PLIF solution.

### 2.3. Film Conditions

In the work presented in this article, the working fluids include deionized water and aqueous glycerol water solution (glycerol concentration 45% by volume), and the experimental conditions cover the range of Re = 16.34~52.54 for water and Re = 1.61~7.43 for the aqueous glycerol water solution. The detailed properties of the working fluids are given in Table 1 below.

With all the properties, the dimensionless groups are determined with the following equations [2].

Reynolds number:

(1) $$Re=\frac{Q/W}{\vartheta_{l}}$$

where *Q* represents the volume flow rate, *W* is the film width, and $\vartheta_{l}$ is the kinematic viscosity of the working fluid. In the following equation, q=Q/W is used to symbolize the unit width flow rate.

Kapitza number:

(2) $$Ka=\frac{\sigma_{l}}{\rho_{l}\vartheta_{l}{}^{4/3}{\left(sin\left(\beta \right)g\right)}^{1/3}}$$

where $\sigma_{l}$ represents the surface tension of the working liquid, $\rho_{l}$ is the density of the working fluid, and *g* represents the gravitational acceleration.

## 3. Experimental Methodology

**Figure 4:**
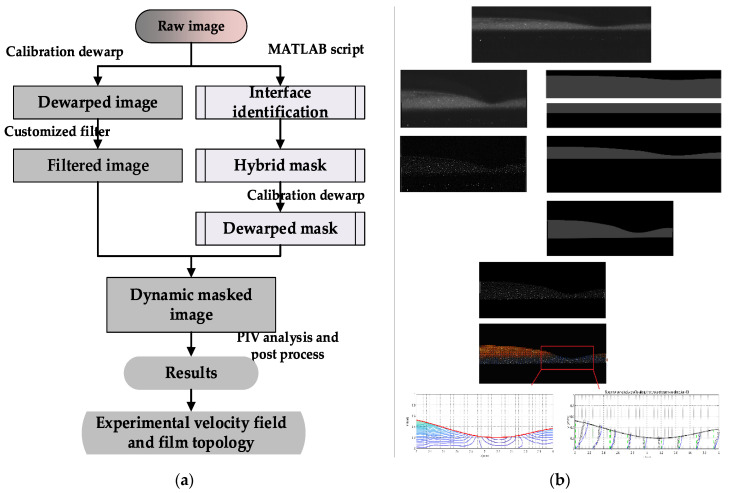
Schematic illustration of PIV/PLIF process. (**a**) PIV/PLIF process. (**b**) PIV/PLIF process example. Each of the procedures in (**a**) corresponds to an image outcome in (**b**) in the order of top to bottom, and then left to right [19].

In this publication, the experimental methodology is essentially the same as that presented previously [19]. However, the interface identification and methodology validation are improved. The elaboration here mainly focuses on the changes, and the details of the remaining methodology can be found in Wang [19]. Figure 4 provides a schematic illustration and an example of the whole set image process.

### 3.1. Image Calibration and Dewarping

With nonuniform refraction index media in the optical path and a high-magnification off-axis image acquisition system, the recorded images are distorted due to nonuniform magnification in different directions, partial defocusing, and imaged plane tilting from the nominal focused plane. Image correction and calibration processes are essential before further investigation and analysis. The corresponding procedures of image distortion correction were accomplished with Dantecs software DynamicStudio by means of multicamera calibration methods with a manual calibration target and pinhole image mode.

### 3.2. Interface Identification with PLIF

The imaged film region was uneven and thin, and complicated image composition has been reported by many researchers. The working fluids used in the experiment were mixed with both the florescent dye and the titanium dioxide particles recorded in the images; therefore, it was necessary to identify the composition of the acquired image prior to further processing.

**Figure 5:**
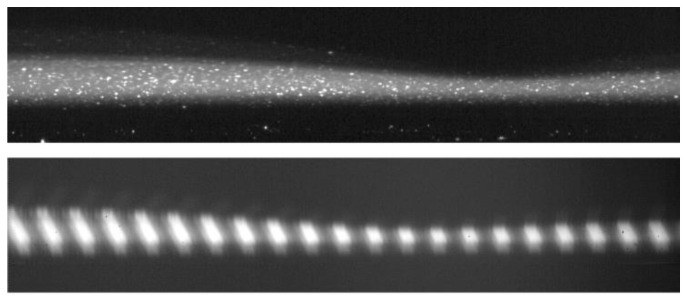
Raw PIV image (**above**) and SPLIF image (**below**). The two images here do not rigorously represent the same state of a capillary wave.

SPLIF, a technique evolved from PLIF, was performed to decompose the acquired image composition. In SPLIF, the planar light sheet is shadowed with a structured plate to form parallel linear illumination, and explicit discrepancies in the fluorescence intensity or orientation emerge after reflection or refraction when light passes through the interfaces [23], which makes it feasible to discern the interfaces and different domains in the image. Figure 5 displays a film SPLIF image, from which it could be inferred that there is a refraction zone above the film–air interface. The bottom of the film domain is estimated to be a projection of the laser plane, the thickness of which is not negligible when the camera is tilted. In addition, the laser sheet thickness is a crucial factor deciding the particle density in the imaged plane and influences the exact velocity profile by the overlay effect in finite volume. In experiments, a thin laser and high particle concentration were adopted to guarantee sufficient particle density with an explicit laser sheet position.

**Figure 6:**
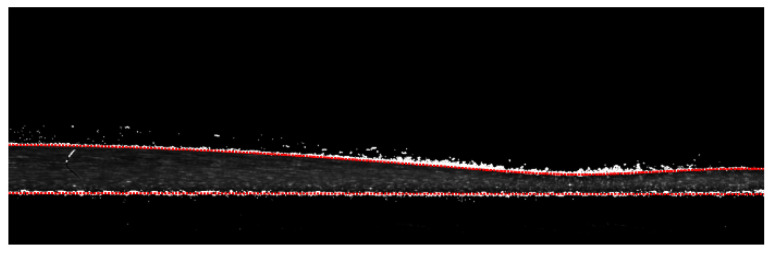
Interface identification example.

With the image constitutions decomposed, it is possible to identify the interfaces according to the greyscale image. The particle images and fluorescence mixing nature sometimes make the results of gradient-based interface identification noisy. Hence, an interface identification method depending on the fluorescence background contribution in image pixel values was applied in image pre-processing. This interface identification method was still accomplished with the convenience of the MATLAB link in DynamicStudio software. Figure 6 shows an example of interface identification, where the white pixels represent pixels falling into the greyscale range and the red lines are the final interface results obtained after fitting.

### 3.3. PIV Method

The adaptive PIV scheme parameters were as follows: maximum interrogation areas (IA) size: 64 × 32; minimum IA size: 32 × 16; and grid step size: 8 × 4. The step size was chosen to better adapt the velocity field grid to the film boundary interface. With the chosen step size, pixel element dimension and optical magnification, the spatial resolution could be inferred to be approximately 25 µm per vector in the cross-stream direction.

During the adaptive adjustment iteratively applied to each IA, the particle density limits the IA size, and the velocity gradient limits the IA shape [24,25,26]. Specifically, the particle density setting limits the lowest particle detection in each IA and the desired number of particles in each IA, which were set to four and eight, respectively, which are relatively low to adapt to the high optical magnification and small investigated zone. For the velocity gradient, the absolute magnitude of each of the four gradients was limited to 0.1, and the combined effect of all four gradients was limited to 0.2, which were the default parameters presented by DynamicStudio.

Finally, the results were tested with a universal outlier detection algorithm [27], peak height validation, and moving average validation (5 × 3 area with acceptance factor 0.1 and iteration 3).

### 3.4. Adaptive PIV Test via Synthetic Particle Images

The quality of the adaptive PIV measurement method was additionally evaluated using synthetic particle images with known properties [28,29]. The considered image effects in synthetic particle images include the particle image diameter, particle density, and motion blur. The synthetic images have dimensions of 512 × 256 pixels. From bottom to top, pixels 1–64 of 256 are left blank as the boundary, and pixels 65–256 are filled with synthetic particle images with a parabolic velocity profile.

**Figure 7:**
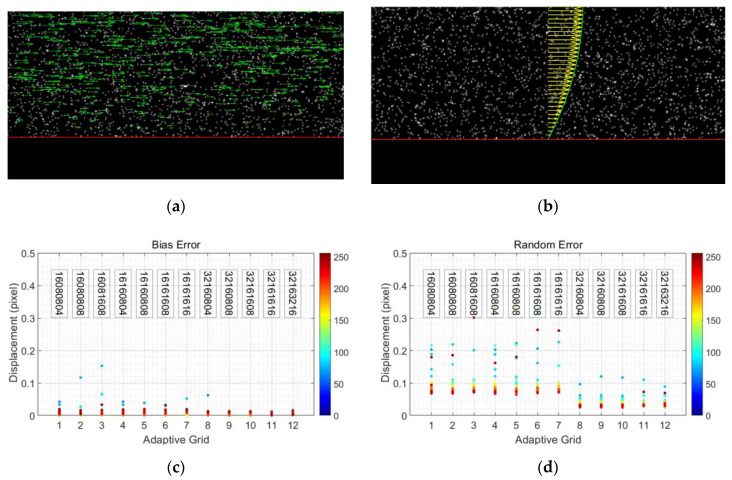
Adaptive PIV test with synthetic particle images: (**a**,**b**) synthetic particle images; (**c**,**d**) test results of bias error and random error, and the color scheme associated with the data points corresponds to the vertical pixel position. Texts in (**c**,**d**) suggested the applied adaptive PIV parameters: the initial 4-digit number were the minimum IA size, and the last 4 represented the grid step size.

Under optimal conditions (particle image diameter ≈ 3 pixels, particle density ≈ 0.02 particles/pixel, no noise, no particle pair loss), the bias error of selected adaptive PIV algorithms is smaller than 0.1 pixels, and the random error is below 0.1 pixels, which might be even more precise and accurate if the lowest position is ignored. As shown in Figure 7, the largest random error and bias error are usually at the edge of the boundary; hence, in the calculation of wall shear stress, the second and third vectors are used instead of the first velocity.

### 3.5. Experimental Methods Validation

#### 3.5.1. Streamwise Velocity Profile Validation via PIV/PLIF, PTV and Nusselt’s Theory

The PIV/PLIF measurement was validated by comparing the experimental PIV/PLIF results with the experimental PTV results and theoretical results under steady smooth film flow conditions. For the theoretical aspects, when Re < Rec [30] (5/4cot(β), approximately 3.4 for β = 20°) film flow falling on an inclined plate is developed, and laminar flow submits to Nusselt’s flat film solution [30]. Then, the theoretical velocity profile could be reconstructed with flow rate and fluid properties. For the PTV parts, it is expected that the PTV results are more accurate and have higher resolution than the PIV results because PTV operates on individual particles instead of a collection of particles [22,31]. In the PTV application for Re = 1.61, f = 0 Hz and Re = 2.22, f = 0 Hz glycerol film flow cases, and determination of the mean velocity profile is achieved by fitting the data with a second-order polynomial function in MATLAB with the curve fitting toolbox using default settings.

The boundaries were recovered from the polynomial, where the zero streamwise velocity suggested that the cross-stream position is the liquid–solid interface and the maximum streamwise velocity corresponds to the liquid–gas interface.

**Figure 8:**
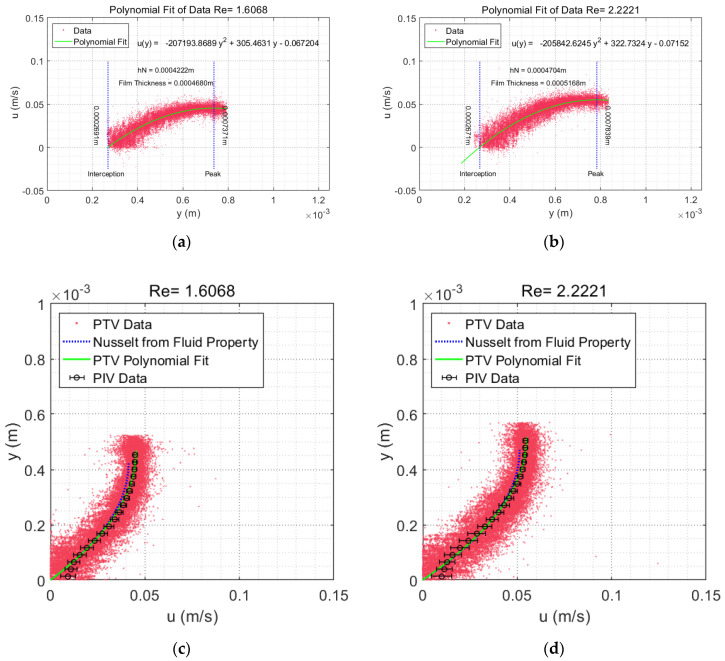
(**a**,**b**) The PTV velocity fitting results and boundary identification for the Re = 1.61, f = 0 Hz and Re = 2.22, f = 0 Hz glycerol film flow cases; the corresponding adjusted *R*^2^ values are 0.8317 and 0.8336, respectively. (**c**,**d**) The velocity profile validation and film thickness validation for the Re = 1.61, f = 0 Hz and Re = 2.22, f = 0 Hz glycerol film flow cases, respectively. For the PIV/PLIF results, horizontal error bars illustrate the standard deviation of the measurement data.

Figure 8c,d illustrates comparisons among the PIV/PLIF experimental results, PTV experimental results and corresponding analytical velocity profiles based on Nusselt’s equation for the Re = 1.61, f = 0 Hz and Re = 2.22, f = 0 Hz glycerol water film flow cases. The comparison showed good agreement among the overall datasets, and a general conclusion could be drawn that the PIV/PLIF film flow experimental results match well with Nusselt’s theoretical prediction for both the film thickness and the velocity profile.

However, the PIV/PLIF velocity in the near-wall region (0–0.1 mm) is biased for the average effect where a strong velocity gradient near the wall exists [31,32], and the profile from the PTV results avoids the wall effect in PIV. Interestingly, above the near-wall region, the PIV/PLIF results and PTV results coalesced perfectly. For the zone near the liquid–air interface, the experimental velocity was larger than Nusselt’s velocity. Compared to the analytical film thickness, PIV/PLIF and PTV slightly overestimated the film thickness.

The velocity difference in the upper region and the film thickness difference might be caused by the following factors: (i) the edge effect in the film spanwise distribution, which makes the film thicker in the middle and thinner near the edge; and (ii) errors from the liquid property measurements, specifically, the viscosity measurement of the glycerol water solution. Even though the PTV scatter plot for the validation achieved a more accurate result, for the instantaneous velocity distributions, the PIV results were more appropriate, because PIV does not evaluate individual particle images, but ensembles of particle images grouped together in IA [22].

#### 3.5.2. Continuity Equation Validation

**Figure 9:**
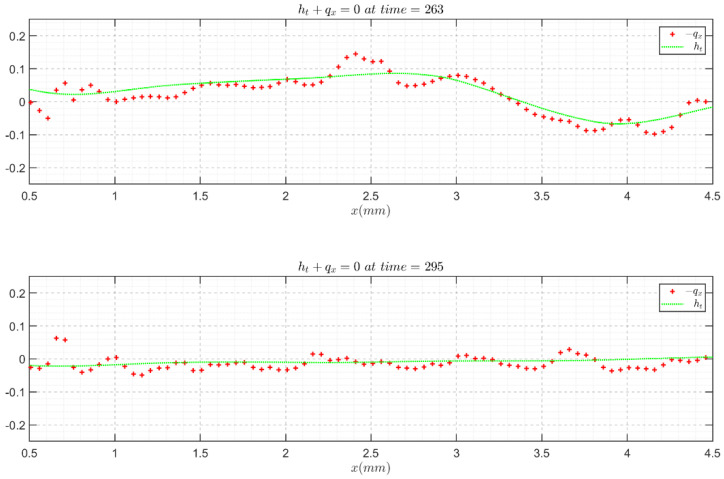
Continuity equation validation with the instantaneous velocity field and film topology for the Re = 41.05, f = 5 Hz water film flow case.

**Figure 10:**
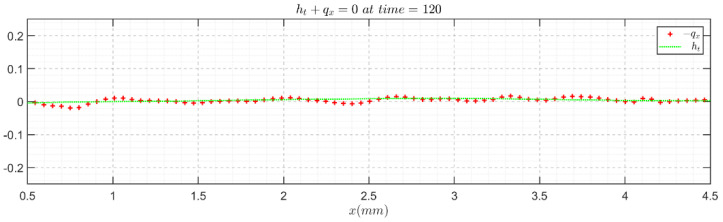

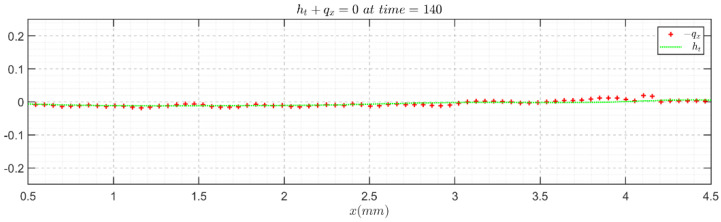
Continuity equation validation with the instantaneous velocity field and film topology for the Re = 7.43, f = 5 Hz glycerol film flow case.

The above validations are mainly based on flat films. To directly check the validity of the instantaneous wavy film velocity field and topology, we resorted to the continuity equation in integral form (Equation (7)) [33], because high-fidelity velocity and topology results would be required to acquire a satisfying reproduction from the experimental outcome. The continuity validation results for Re = 41.05, f = 5 Hz and Re = 7.43, f = 5 Hz are presented in Figure 9 and Figure 10, respectively, as examples, in which the negative value of the flow rate streamwise derivative generally agrees well with the experimental thickness time derivative, assuring the fidelity and accuracy of the PIV/PLIF measurement techniques.

(3) ∂h/∂t+∂q/∂x=0

## 4. Results and Discussions

In this section, we present the results and discussions based on the experimental velocity field, topologies, and their derivatives. Consecutive images of film flow through the imaged zone of the test section were acquired for each case. It is impossible to list all the moments in this article; therefore, the film flow characteristics are presented only with limited snapshots. Images of the same case and at the same time indicate the same condition, which could be used to correlate for better comparison.

### 4.1. Model-Based Velocity Field Reconstruction

Our goal of this research was to establish a film model validation method specific to the results of a comparison between the model-based reconstructed velocity profiles and experimental velocity profiles. The velocity field data have a high temporal resolution (0.0005 s) and high spatial resolution (approximately 0.025 mm per vector in the cross-stream direction); therefore, it is reasonable to use experimental flow variables and their derivatives for reconstruction of the velocity field with model velocity profile assumptions.

The velocity profiles of the film models used in the velocity field reconstruction include Nusselt’s velocity profile, Kapitza’s velocity profile, the streamwise velocity profile in the two equations of the first-order weighted residuals model (1st WRM) and the second-order regularized model (RM). The streamwise velocity formulas used are given below:

(1) The parabolic velocity profile in Nusselt’s theory [34], u(y):
  (4) $$u\left(y\right)=\frac{gh^{2}\cos\left(\beta \right)}{2\mu }\left[\frac{2y}{h}-{(\frac{y}{h})}^{2}\right]$$
  where *g* is the acceleration due to gravity, h is the local film thickness, *ρ* represents the fluid density, μ stands for the dynamic viscosity, and β is the inclination angle [34]. With the formula given, a parabolic profile could be readily constructed with local film heights.
(2) Self-similar velocity profile assumption based on local film thickness and flow rates, which is given by Kapitza et al. In this paper, we refer to that equation as Kapitza’s velocity profile. This velocity profile corresponds to a first-order correction of Nusselt’s theory, which could adapt the velocity to the deformations of the free surface and changes in the flow rate [35,36,37].
  (5) $$u\left(y\right)=3\frac{q}{h}\left[\frac{y}{h}-\frac{1}{2}{(\frac{y}{h})}^{2}\right]$$
  From Equation (5), a self-similar velocity can be acquired with an elementary experimental velocity field and film topology.
(3) The streamwise velocity profile in the first-order weighted residuals model is a series expansion, and the profile finally adopted in the 1st WRM is given below:
  (6) $$u\left(y\right)=3\frac{q}{h}f_{0}+a_{1}(-\frac{2}{5}f_{0}+f_{1}-\frac{1}{3}f_{2})+a_{3}(\frac{8}{35}f_{0}-\frac{4}{3}f_{2}+f_{3}-\frac{1}{5}f_{4})$$
  (7) $$a_{1}=3Re\left[-\frac{1}{2}h\partial_{t}q-\frac{1}{2}h\partial_{x}\left(\frac{q^{2}}{h}\right)\right],$$
  (8) $$a_{3}=3Re\left[-\frac{3}{20}h^{3}q\partial_{x}\left(\frac{q}{h^{3}}\right)\right],$$
  (9) $$f_{j}={\bar{y}}^{j+1}-\frac{j+1}{j+2}{\bar{y}}^{j+2},\;j=0,1,2,3,4,$$
  where the test functions $f_{j}$, amplitudes $a_{j},\;j=0,1,2,3,4$ and reduced variable $\bar{y}=y/h$ formed the velocity profiles.
(4) The second-order regularized model reduced the four-equation first-order weighted residual model (2nd WRM) by eliminating the independence of $s_{1}$ and $s_{2}$ [2]. The streamwise velocity profile in the regularized model is given by the same formula as the 2nd WRM assumption.
  (10) $$u\left(y\right)=\frac{3}{h}\left(q-s_{1}-s_{2}\right)F_{0}+45\frac{s_{1}}{h}F_{1}+210\frac{s_{2}}{h}F_{2}$$
  (11) $$F_{0}=\bar{y}-\frac{1}{2}{\bar{y}}^{2},$$
  (12) $$F_{1}=\bar{y}-\frac{17}{6}{\bar{y}}^{2}+\frac{7}{3}{\bar{y}}^{3}-\frac{7}{12}{\bar{y}}^{4},$$
  (13) $$F_{2}=\bar{y}-\frac{13}{2}{\bar{y}}^{2}+\frac{57}{4}{\bar{y}}^{3}-\frac{111}{8}{\bar{y}}^{4}+\frac{99}{16}{\bar{y}}^{5}-\frac{33}{32}{\bar{y}}^{6},$$
  (14) $$s_{1}=3Re\left(\frac{1}{210}h^{2}\partial_{t}q-\frac{19}{1925}q^{2}\partial_{x}h+\frac{74}{5775}hq\partial_{x}q\right),$$
  (15) $$s_{2}=3Re\left(\frac{2}{5775}q^{2}\partial_{x}h-\frac{2}{17325}hq\partial_{x}q\right),$$
  where $F_{j},j=0,1,2$ is an orthogonal basis that is constructed through the Gram–Schmidt orthogonalization procedure, and $s_{1},s_{2}$ are inertia corrections for the velocity distribution.

Reconstructed streamwise velocity profiles and velocity fields with different numerical models were compared to experimental results. However, the four equations of the 2nd WRM need four independent variables (*h*, *q*, $s_{1}$, and $s_{2}$) to support the reconstruction, among which the independent $s_{1}$ and $s_{2}$ are beyond this experimental data range. Hence, the 2nd WRM was not adopted in velocity profile construction and comparison.

### 4.2. Comparison of Instantaneous Velocity Profiles

In the presented comparisons, the experimental instantaneous velocity profiles were plotted against different model-reconstructed velocity profiles, and in addition to the velocity profile, different time slices are listed to include typical zones in solitary waves.

**Figure 11:**
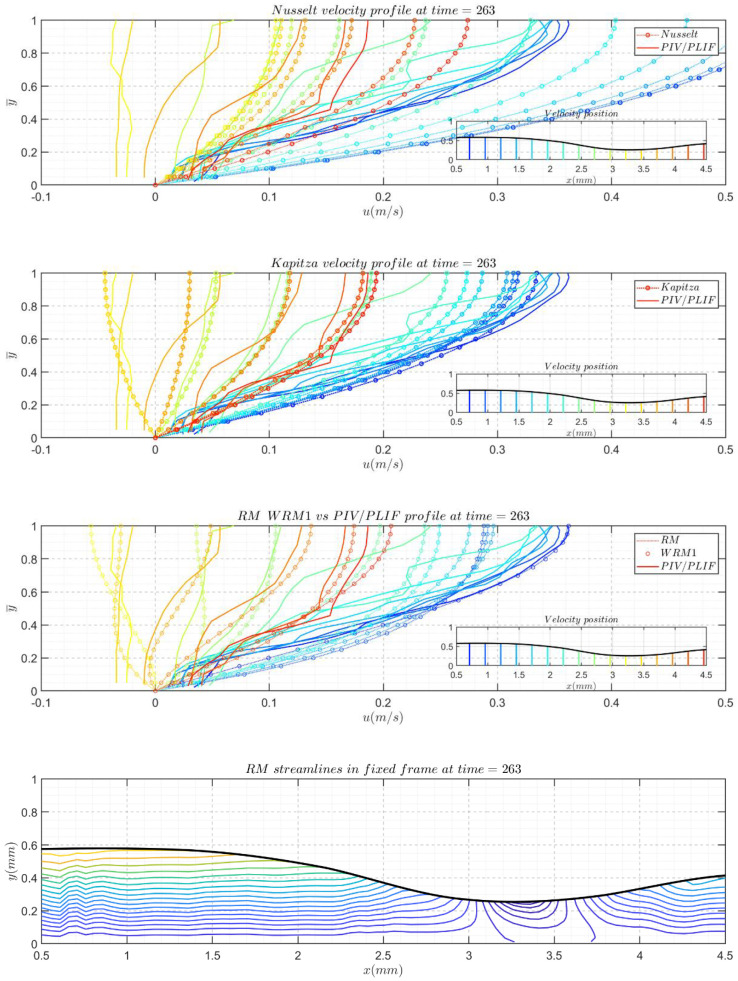

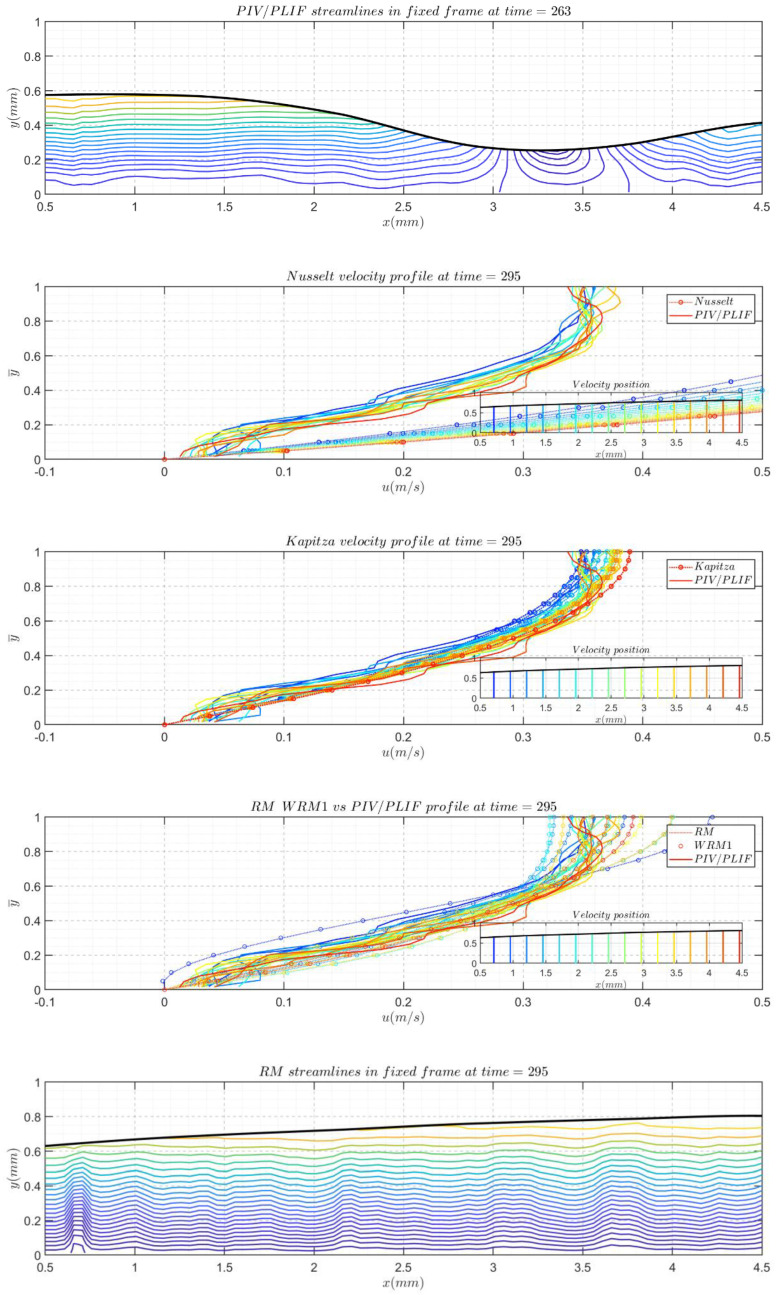

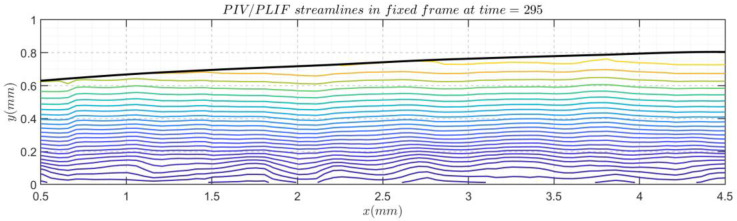
Comparison between the experimental instantaneous velocity profiles and corresponding model-reconstructed velocity profiles for the Re = 41.05, f = 5 Hz water film flow case.

**Figure 12:**
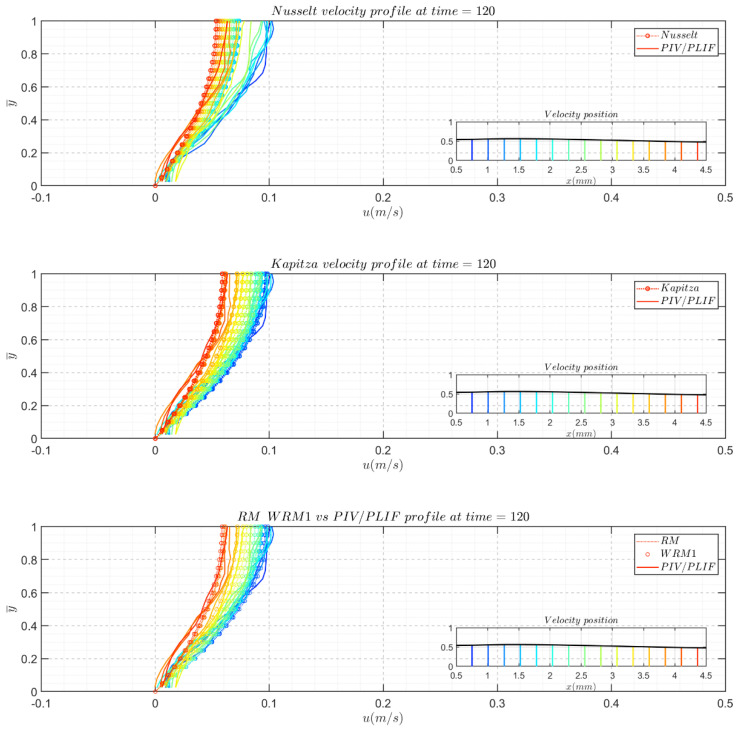

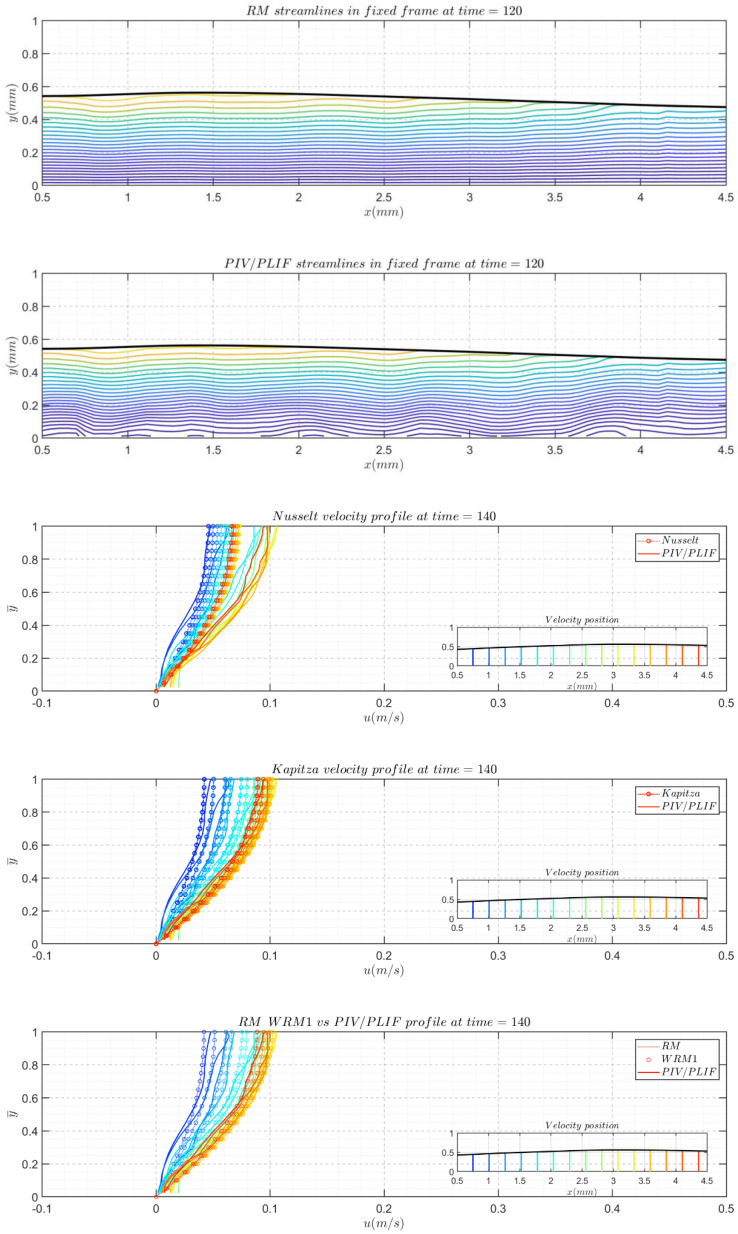

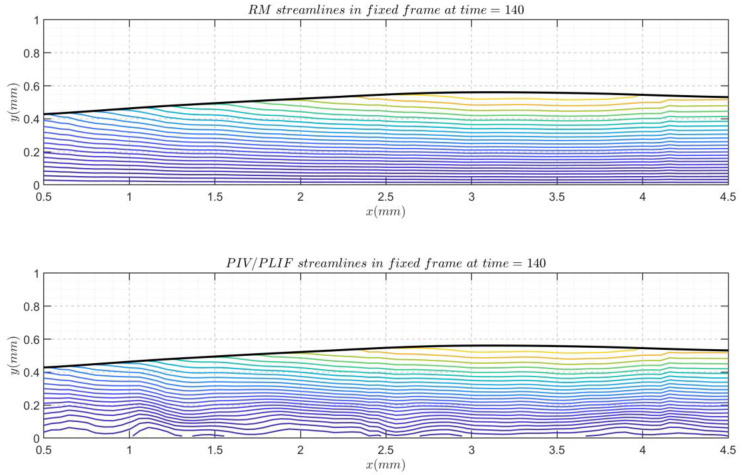
Comparison between the experimental instantaneous velocity profiles and the corresponding model-reconstructed velocity profiles for the Re = 7.43, f = 5 Hz glycerol film flow case.

For each snapshot, the velocity profiles are plotted against the model-reconstructed profiles in three subfigures. In each subfigure of the profile comparison, the *y*-axis represents $\bar{y}$, which is normalized by the local film thickness to unity, and different colors indicate different profile positions. Moreover, the free surface of the film determined from PIV/PLIF images is displayed in pictograms. They do not cover the entire region because the boundary might introduce some drawbacks when calculating derivatives. In pictograms, different velocity profile positions are highlighted with correlating colored lines to show their streamwise positions in the film topologies. The streamwise velocity field streamlines are also given for the RM results and experimental PIV/PLIF results to present a more direct comparison. However, in the following section, only some of the canonical moment snapshots are presented here. Figure 11 and Figure 12 present the typical responses of the Re = 41.05, f = 5 Hz and Re = 7.43, f = 5 Hz cases to disturbances. Conclusions can be drawn after the careful examination of Figure 11 and Figure 12:

Nusselt’s profile underestimated the velocity magnitude for Re = 7.43, f = 5 Hz and overestimated the velocity magnitudes in the case of Re = 41.05, f = 5 Hz. Except in the fast-changing zones, the fronts of the main hump and capillary wave crests were similar.

Kapitza’s velocity profile, the 1st WRM velocity profile, and the RM velocity profile work very well for the Re = 7.43, f = 5 Hz glycerol cases because Kapitza’s velocity profile (Equation (10)) correlates with the first term of the 1st WRM (Equation (11)) and RM (Equation (15)) velocity profiles, and further correction did not yield a vital difference in the aspects of the velocity profile based on flow rates and film thickness for the glycerol water solution cases.

The 1st WRM and RM are both two-equation models; the RM is reduced from the four-equation WRM, however these two models currently give very similar velocity fields. From the reconstructed velocity profiles, no cases or data in this study could induce significant differences.

Compared to the experimental velocity field, the 1st WRM and RM fields were not able to precisely describe the actual velocity field in the film flow for Re = 41.05, f = 5 Hz in front of the main hump and the first capillary wave troughs.

## 5. Summary

In this film flow experiment, the instantaneous velocity field and film topology of disturbed falling liquid films were measured simultaneously using the PIV/PLIF technique to determine the hydrodynamic characteristics of excited thin film flows. The applied experimental measurement method could provide a high resolution in both the temporal dimension and the spatial dimension. An experimental validation method for the film flow model velocity field was established by comparing the experimental velocity fields and constructed velocity fields based on the model and elementary experimental data.

An experimental methodology validation was performed, mainly by streamwise velocity profile and film thickness comparisons; experimental PIV/PLIF results were compared to Nusselt’s solution and experimental PTV results. A cross comparison suggests the overall accuracy of the experimental PIV/PLIF results despite the inherent limitations of PIV and uncertainties in the determined flow conditions. Owing to the high temporal and spatial resolution, creditable derivatives are derived. The integral continuity equation is reproduced, which validates the instantaneous velocity field.

Based on the film response to the inlet disturbance, solitary wave cases were chosen to exhibit the flow field hydrodynamic characteristics. To validate the film models, model-predicted velocity fields were calculated based on the experimental flow rate, film thickness and their derivatives; the validated models included Nusselt’s theory, Kapitza’s theory, and the 1st WRM and RM models. After a comparison between the experimental results and reconstructed velocity profiles, the following conclusions could be drawn: (i) under the majority of conditions, Nusselt’s theory underestimates the velocity in the glycerol water solution but overestimates the velocity in the water film cases, except for rapidly changing parts such as capillary wave troughs. (ii) Kapitza’s model could be referred to as the first term of the velocity profile, which already performs well to depict the flow field for the glycerol film cases. For the water film cases, the Kapitza model failed to produce such complex transient velocity fields. (iii) The 1st WRM was very close to the 2nd RM with identical flow rates and surface boundaries for all the cases considered in this research. The 1st WRM could reconstruct the velocity field well for most zones, except the front of the main hump and the first capillary wave troughs, where the velocity profiles were rapidly changing. Our research, experimental methods and experimental data should be of interest for film-related experiments and simulation studies.

## Figures and Tables

**Table 1:** Working fluid properties.

Working Fluid	*ρ* kg/m^3^	μ kg/(m·s)	σ N/m
Deionized water	0.998 × 10^3^	10.3 × 10^−4^	70.7 × 10^−3^
Glycerol water solution	1.120 × 10^3^	81.0 × 10^−4^	65.4 × 10^−3^

## Data Availability

The data presented in this study are available on request from the corresponding author.
